# Bacterial Vaginosis and the Natural History of Human Papillomavirus

**DOI:** 10.1155/2011/319460

**Published:** 2011-08-16

**Authors:** Caroline C. King, Denise J. Jamieson, Jeffrey Wiener, Susan Cu-Uvin, Robert S. Klein, Anne M. Rompalo, Keerti V. Shah, Jack D. Sobel

**Affiliations:** ^1^Division of Reproductive Health, National Center for Chronic Disease Prevention and Health Promotion, Centers for Disease Control and Prevention, NCCDPHP, DRH, 4770 Buford Highway, Mailstop K-34, Atlanta, GA 30341, USA; ^2^Department of Obstetrics and Gynecology, Alpert Medical School of Brown University, Providence, RI 02912, USA; ^3^Department of Medicine, Mount Sinai School of Medicine, New York, NY 10029, USA; ^4^Division of Infectious Diseases, School of Medicine, Johns Hopkins University, Baltimore, MD 21205, USA; ^5^Department of Molecular Microbiology and Immunology, Johns Hopkins Bloomberg School of Public Health, Baltimore, MD 21205, USA; ^6^Division of Infectious Diseases, Wayne State University School of Medicine, Detroit, MI 48201-1998, USA

## Abstract

*Objective*. To evaluate associations between common vaginal infections and human papillomavirus (HPV). *Study Design*. Data from up to 15 visits on 756 HIV-infected women and 380 high-risk HIV-uninfected women enrolled in the HIV Epidemiology Research Study (HERS) were evaluated for associations of bacterial vaginosis, trichomoniasis, and vaginal *Candida* colonization with prevalent HPV, incident HPV, and clearance of HPV in multivariate analysis. *Results*. Bacterial vaginosis (BV) was associated with increased odds for prevalent (aOR = 1.14, 95% CI: 1.04, 1.26) and incident (aOR = 1.24, 95% CI: 1.04, 1.47) HPV and with delayed clearance of infection (aHR = 0.84, 95% CI: 0.72, 0.97). Whereas BV at the preceding or current visit was associated with incident HPV, in an alternate model for the outcome of incident BV, HPV at the current, but not preceding, visit was associated with incident BV. *Conclusion*. These findings underscore the importance of prevention and successful treatment of bacterial vaginosis.

## 1. Introduction

Human papillomavirus (HPV) is the most common sexually transmitted infection (STI) among young adult women, and it plays a critical role in the development of cervical cancer [1, 2]. Biologic susceptibility to HPV acquisition and immune competence for clearance of an HPV infection could be affected by common, treatable vaginal infections that disrupt the intricately balanced vaginal ecosystem and its innate protective mechanisms against infection and disease. Bacterial vaginosis and trichomoniasis are associated with high levels of anaerobic microorganisms and their byproducts, that can damage vaginal epithelium, degrade cervical mucus, and cleave immunoglobulin A [3–6]. Despite the similar effects of bacterial vaginosis and trichomoniasis on the vaginal environment, there is increasing evidence only for an association between HPV and bacterial vaginosis, albeit of unknown directionality [7–9]. Bacterial vaginosis and trichomoniasis are categorized, along with vulvovaginal candidiasis, under the somewhat misnomer of vaginitis. Unlike bacterial vaginosis and trichomoniasis, studies on vulvovaginal candidiasis are complicated by its lack of a clear laboratory definition, since the etiologic agent may exist as part of the commensal microbiota in healthy women. *Candida albicans* is the most prevalent species in most cases of asymptomatic colonization and vulvovaginal candidiasis; however certain species of *Candida *are more pathogenic than others and induce hyphae formation and have enhanced proteolytic activity and antigen modulation, enabling *Candida* to penetrate the mucosal surface and induce mucosal swelling, erythema, and exfoliation of cells [10–12]. The role of vaginal infections in HPV pathogenesis has been evaluated in primarily cross-sectional studies and has yielded conflicting results [8, 9, 13–19]. The purpose of the current study was to evaluate associations for vaginal infections with prevalent HPV, incident HPV, and clearance of HPV in a large, prospective cohort of HIV-infected and high-risk, HIV-uninfected women.

## 2. Materials and Methods

The HIV Epidemiology Research Study (HERS) was a longitudinal multi-site investigation of the biological, psychological, and social effects of HIV infection among US women [20]. Briefly, from April 1993 through January 1995, 871 HIV-infected women and 439 high-risk HIV-uninfected women were enrolled at four study sites: Bronx, NY; Baltimore, MD; Detroit, MI; and Providence, RI. Institutional review boards at each participating medical center and at the Centers for Disease Control and Prevention approved the study protocol. All participants gave informed consent and agreed to HIV testing and counseling. Women aged 16–55 years were eligible for participation if they had a history of injection drug use or high-risk sexual behavior, were fluent in either English or Spanish, and if HIV-seropositive, reported no history of clinical AIDS-defining conditions. At each study site, HIV-seronegative women were frequency matched to HIV-seropositive women on HIV status (1 : 2 ratio) and HIV risk behavior (1 : 1, sexual behavior to injection drug use). Women completed up to 15 study visits scheduled 6 months apart that consisted of an interview, physical and pelvic examination, and blood, urine, oral, and cervicovaginal specimen collection. 

HPV testing used polymerase chain reaction (PCR) to amplify virus obtained by cervicovaginal lavage [21]. The amplified product was identified by hybridization with a generic HPV probe and probes specific to 26 HPV types: 6, 11, 16, 18, 26, 31, 33, 34, 39, 40, 42, 45, 51, 52, 53, 54, 55, 56, 58, 59, 66, 68, 73, 82, 83, and 84. Samples with missing (*n* = 17) or negative (*n* = 72) test results for hybridization with the control, B-globin probe were considered of unsatisfactory quality, and the HPV results were set to missing. 

For the purposes of the current analysis, visit-specific prevalent HPV infection was defined by detection of any HPV DNA by general probe at a given visit. Incident infection was defined as detection of a new type of HPV not present at any prior visit. Thus, HPV type-specific negative women at enrollment were at risk for infection with the respective HPV type. Women who were HPV-negative by all type-specific probes and generic probe prior to testing positive by generic probe only (i.e., untypable infection) were classified as having incident HPV at that visit. For the evaluation of time to HPV clearance, women contributed event times for each incident HPV type. Time to clearance was defined as the interval from the type-specific incident visit to the first of two consecutive type-specific negative visits. The flagging negative visit was required to avoid misclassification from fluctuating detection of HPV [22]. HPV infections that were followed by a single HPV-negative, final study visit were censored at that negative visit. HPV infections that were positive at the last study visit had 6 months added to ensure comparability of assumptions about clearance of HPV infection for censored and noncensored observations. HPV infections spanning a single missing visit were assumed to persist over the unobserved period. 

Each vaginal infection was defined using standard criteria. Presence of bacterial vaginosis was determined by Nugent scoring of a gram-stained slide [23]. A Nugent score of 7 to 10 defined bacterial vaginosis, whereas a score of 0 to 6 defined normal-intermediate vaginal flora. For visits without a Nugent score (<1%), bacterial vaginosis was defined by modified Amsel's criteria [23]. Presence of vaginal *Candida *colonization was based on a positive culture for *Candida* species or detection of yeast or pseudohyphae on gram stain. Presence of trichomoniasis was determined by detection of trichomonas on wet mount (all visits) or on culture (visits 4 and above). 

Women were eligible for the current analysis if they had baseline data on HPV DNA and bacterial vaginosis, vulvovaginal candidiasis, or trichomoniasis test results (*n* = 1256). Of these women, 108 who did not have a cervix at baseline and one woman who had cervical treatment and a hysterectomy within 6 months before and after enrollment, respectively, were excluded. For simplicity, an additional 11 women who seroconverted to HIV-seropositive during follow-up were excluded, leaving 1136 subjects for this analysis. Person-time was censored at the date of cervical treatment (e.g., excisional biopsy, cryotherapy) or hysterectomy for 111 subjects. 

Potential confounders of the relationship between vaginal infections and prevalent HPV, incident HPV, and clearance of HPV were selected based on the literature and a proposed causal directed acyclic diagram (not shown) used for identification of sufficient sets of confounders [24]. The *a priori* model for prevalent HPV and incident HPV included sexual risk factors (new or multiple male sex partners and frequency of condom use), illicit drug use, cigarette smoking, use of hormonal contraceptives, immunologic predisposing factors (HIV status, CD4 count, and viral load), and demographic indicators for HPV prevalence in the USA (age, race, and socioeconomic status) [1, 25]. *A priori* models for time to HPV clearance also included a covariate indicating coinfection with another HPV type within the type-specific duration and its cancer risk classification. All statistical models included the study design variables: study site and risk behavior cohort (sexual or injection drug use). In addition, models included a covariate for visit number, year of visit, or year of HPV onset to account for period effects.

In addition to the variables selected *a priori*, other factors related to cervicovaginal health were evaluated in bivariate and multivariable analysis using SAS versus 9.2 (SAS Institute Inc., Cary, NC). These factors included potential hormonal influences (e.g., pregnancy, postmenopausal state, and estrogen replacement therapy), sexual behavior (e.g., sexual frequency), genital tract infections (e.g., herpes simplex virus-2 serostatus at baseline—culture and PCR testing for chlamydia and gonorrhea ceased after the third study visit due to very low frequency in this population), and other risk behaviors or conditions (e.g., alcohol drinking and hepatitis C serostatus at baseline). The importance of a covariate was evaluated in terms of the causal directed acyclic graph, collinearity with other covariates, and its coefficient magnitude, statistical significance, and impact on the effect estimates for vaginal infections upon addition to and removal from the statistical model. 

A generalized linear mixed model with a random intercept and a slope based on a toeplitz matrix was used to model associations between current vaginal infections and prevalent HPV while accounting for between-subject variation and repeated measures correlation. To evaluate associations between vaginal infections at the preceding visit and incident HPV, as well as to explore the temporal relationship between HPV and bacterial vaginosis, the case-crossover analytic approach was used. The case-crossover approach is useful for identifying associations between recent, time-varying exposures and an incident outcome while adjusting for all time-independent factors by comparing each woman's case (e.g., incident HPV) visits to her own noncase visits. To evaluate temporality, separate case-crossover models were run for incident HPV and incident bacterial vaginosis that were adjusted for a few shared time-dependent predictors: a combined indicator for HIV status and CD4 category (with 4 categories: HIV-negative and HIV-positive with CD4 > 500, CD4 200–500, and CD4 < 200) and self-reported crack or cocaine use, new or multiple sex partners, and consistency of condom use since the prior study visit.

Analyses of time to clearance of a type-specific HPV infection used the Wei, Lin, and Weissfeld extended Cox model. Hazard ratios less than 1.0 indicate lower clearance rates (i.e., longer duration of infection). Vaginal infections, and most of the covariates, were modeled as time-dependent variables allowing the instantaneous effects to vary with changes in the time-dependent variables. 

Upon finalizing the main effects models, modification of the effect estimates for vaginal infections across levels of biologically relevant covariates (e.g., HIV status, concurrent vaginal infections) was evaluated for magnitude and significance (*P* < 0.15) using the Wald and Likelihood Ratio tests. Due to effect measure modification of the association between certain vaginal infections and HPV outcomes by HIV status, tabular results are presented separately for HIV-infected and HIV-uninfected women. 

## 3. Results

At baseline, HIV-infected women (*n* = 756) were similar to HIV-uninfected women (*n* = 380) in age, number of live births, cigarette smoking status, and prevalence of bacterial vaginosis, *trichomonas vaginalis*, *Chlamydia trachomatis*, and* Neisseria gonorrhoeae *infections (Table 1). The HIV-uninfected women were more likely to have recently used crack cocaine or injection drugs and had multiple sex partners in the prior 6 months. The HIV-infected women were more likely to be of African American race, of lower educational level, and seropositive for hepatitis C and herpes simplex 2. HIV-infected women were also more likely to be vaginally colonized with *Candida* species and have detectable HPV. The prevalence of HPV among HIV-infected women (64%) was over twice that of HIV-uninfected women (29%), and HIV-infected women were more likely to be coinfected with multiple HPV strains (Table 1). However, among women with HPV, the proportion with untypable HPV infections was greater among HIV-uninfected women (30.3%) than among HIV-infected women (20.3%). At enrollment, most of the HIV-infected women (83%) had CD4 counts above 200 cells/*μ*L. Enrollment was completed before the onset of the highly active antiretroviral therapy (HAART) era, so none of the women were on HAART regimens at baseline. However, one-third of the HIV-infected women were taking antiretrovirals.

The prevalence of HPV infection at visits 1–15 ranged from 38% to 56% (49–68% for HIV-infected women and 12–32% for HIV-uninfected women). Longitudinal, multivariable analysis for prevalent HPV demonstrated a strong association with HIV status and immunodeficiency. HIV-infected women with CD4 cell counts below 200 cells/*μ*L had greater odds for prevalent HPV compared to HIV-infected women with CD4 counts above 500 cells/*μ*L (adjusted odds ratio (aOR) = 1.53, 95% CI: 1.25, 1.86). There was no modification of the odds ratio for bacterial vaginosis on prevalent HPV by HIV status (Table 2). In a model adjusted for a combined indicator of HIV status and CD4 group, bacterial vaginosis was significantly associated with increased odds for prevalent HPV among all HERS women (aOR = 1.14, 95% CI: 1.04, 1.26). HIV status significantly modified the associations among trichomoniasis, vaginal *Candida *colonization, and HPV prevalence. In a model for HIV-uninfected women, there was no significant association for trichomoniasis or vaginal *Candida *colonization with prevalent HPV (Table 2). However, among HIV-infected women, there was a significant interaction between trichomoniasis and vaginal *Candida* colonization: trichomoniasis was significantly associated with decreased odds for prevalent HPV among women without vaginal *Candida *colonization, and vaginal *Candida *colonization was significantly associated with increased odds for prevalent HPV among women with trichomoniasis (Table 2). When vaginal *Candida* colonization was removed from the model, trichomoniasis remained significantly associated with decreased odds for prevalent HPV among the HIV-infected women (aOR = 0.85, 95% CI: 0.73, 0.98). In contrast, when trichomoniasis was removed from the model, vaginal *Candida* colonization was not associated with prevalent HPV (aOR = 1.02, 95% CI: 0.92, 1.12).

Incident HPV infections occurred at 14% of follow-up visits (17.4% among HIV-infected women and 7.5% among high-risk, HIV-uninfected women). There was no modification of the odds ratio for prior bacterial vaginosis on incident HPV by HIV status (Table 3). In a model adjusted for a combined indicator of HIV status and CD4 group, bacterial vaginosis at the preceding visit was significantly associated with 24% increased odds for incident HPV among all HERS women (aOR = 1.24, 95% CI: 1.04, 1.47) (Table 3). Bacterial vaginosis at the current visit was similarly associated with incident HPV (aOR = 1.25, 95% CI: 1.05, 1.48). In contrast, a case-crossover analysis for the outcome of incident bacterial vaginosis found that HPV at the current visit (aOR = 1.46, 95% CI: 1.12, 1.90), but not at the preceding visit (aOR = 1.05, 95% CI: 0.80, 1.37), was predictive of incident bacterial vaginosis among all women. Trichomoniasis and vaginal *Candida* colonization was not significantly associated with incident HPV, regardless of timing of vaginal infection relevant to HPV infection (Table 3). 

The majority of incident HPV infections cleared over subsequent study visits (63.1% clearance among HIV-infected women and 74.8% among HIV-uninfected women). Among HIV-infected women, those with CD4 counts below 200 cells/*μ*L were half as likely to clear an HPV infection as those with CD4 counts above 500 cells/*μ*L (adjusted hazards ratio (aHR) = 0.51, 95% CI: 0.40, 0.64). HIV status and CD4 category did not significantly modify the hazard ratios for vaginal infections with HPV clearance. However, there was a statistical interaction between bacterial vaginosis and vaginal *Candida *colonization (Table 4). Among all HERS women with vaginal *Candida *colonization, bacterial vaginosis was associated with a lower rate of HPV clearance (aHR = 0.65, 95% CI: 0.51, 0.83), and vice versa, among women with bacterial vaginosis, vaginal *Candida *colonization was associated with delayed HPV clearance (aHR = 0.73, 95% CI: 0.58, 0.93). When vaginal *Candida *colonization was removed from the model, bacterial vaginosis remained significantly associated with delayed clearance of HPV among all HERS women (aHR = 0.84, 95% CI: 0.72, 0.97). In contrast, when bacterial vaginosis was removed from the model, vaginal *Candida* colonization was not associated with HPV clearance (aHR = 0.93, 95% CI: 0.81, 1.08). We did not detect a significant association between trichomoniasis and time to clearance of HPV infection, regardless of HIV status (Table 4).

## 4. Comment

Consistent with a recent meta-analysis of primarily cross-sectional studies, bacterial vaginosis was associated with increased odds for prevalent HPV among women in HERS [7]. Furthermore, in this large, prospective study, bacterial vaginosis was associated with increased odds for incident HPV and delayed clearance of HPV. The former finding is consistent with a reported increase in HPV incidence among women with bacterial vaginosis in the Women's Interagency HIV Study (WIHS) [9]. Prior attempts to decipher the temporal relationship between bacterial vaginosis and HPV yielded conflicting findings [8, 9]. In this study, bacterial vaginosis, whether considered at the preceding or current visit, was associated with incident HPV, whereas, in an alternate multivariable model for the outcome of incident bacterial vaginosis, only HPV at the current visit was significant. These findings are in accord with biologic plausibility since, unlike most cervical HPV infections, bacterial vaginosis causes major changes in the local environment leading to degradation of innate defenses. 

While trichomoniasis infection was not significantly associated with HPV outcomes among HIV-uninfected women in HERS, HIV-infected women with trichomoniasis had decreased odds for prevalent HPV. A similar finding was reported from a WIHS analysis which combined 1736 HIV-infected and 493 HIV-uninfected women into one analytic group [9]. In that study, detection of trichomoniasis on wet mount was associated with lower prevalence, increased incidence, and shorter duration of HPV infection. In the current study, we did not detect a significant effect of trichomoniasis on incident HPV or clearance of infection; however, this may have been due to lower statistical power. Smaller studies among only HIV-uninfected women are more consistent with our findings among the HIV-uninfected women in suggesting absence of an association between trichomoniasis and prevalent, incident, or persistent HPV [14, 15, 18]. The apparent difference by HIV status in associations for trichomoniasis with HPV prevalence and duration may be attributable to the strong effect of HIV on duration of HPV infection relative to the minimal, if any, effect of HIV on duration of trichomoniasis. Explicitly, as the duration of HPV infection increases, the proportion of HPV-positive visits that have trichomoniasis present decrease, giving the appearance of a protective association. 

The presence of vaginal *Candida* colonization alone was not significantly associated with HPV outcomes; however when concurrent with bacterial vaginosis or trichomoniasis, it negatively impacted their effects on certain HPV outcomes. The decreased odds for prevalent HPV among women with trichomoniasis was not observed among women with vaginal *Candida *colonization, and the association between bacterial vaginosis and delayed clearance of an HPV infection was strongest among women with vaginal *Candida* colonization. Standard antibiotic treatment for bacterial vaginosis or trichomoniasis may precipitate a vaginal yeast infection, and a high incidence of asymptomatic and symptomatic vulvovaginal yeast infection has been reported in patients with recurrent bacterial vaginosis [27]. Therefore, concurrent vaginal *Candida *colonization may indicate more severe or difficult-to-treat bacterial vaginosis or trichomoniasis. 

The HERS cohort, two-thirds of which are HIV-infected, represents a population at increased risk for vaginal infections and HPV. As such, there are two primary concerns regarding these study's findings: (1) the potential dependency of the results on HIV disease and (2) the generalizability of the results to the general population of women. Several methods were used to reduce potential confounding of the relationships between vaginal infections and HPV by HIV-associated factors: (1) by design, HIV-infected women were frequency matched at enrollment to HIV-uninfected women on sexual and injection drug use behavior, (2) self-reported risk behavior between study visits was included in multivariable analyses, (3) models among all HERS women were evaluated for statistical interaction with a composite variable of HIV status and CD4 count, and (4) separate models were built for HIV-infected and HIV-uninfected women, with the former adjusted for CD4 category and logarithm of viral load. Despite these efforts, disentangling the interrelationships between HIV disease and concurrent vaginal infections was limited by decreased statistical power to detect significant associations among HIV-uninfected women separately. While the HERS cohort is a population at high-risk for genital tract infections, the primary findings involve two infections for which the general female population is also at high risk: bacterial vaginosis and HPV. Moreover, there is mounting evidence for an association between bacterial vaginosis and HPV across diverse study populations [7]. 

This is the second large, prospective study among HIV-infected and HIV-uninfected women to report consistent findings for bacterial vaginosis with increased prevalent and incident HPV [9]. In the current study, bacterial vaginosis was also associated with delayed clearance of HPV infection. While there is mounting evidence for an association between bacterial vaginosis and HPV, a temporal association has remained unclear [7–9]. In the current study, we found that bacterial vaginosis, whether considered at the preceding or current study visit, was associated with incident HPV, whereas HPV at the current, but not preceding, visit was associated with incident bacterial vaginosis. In summary, the current study suggests bacterial vaginosis may predispose a woman to HPV infection and delayed HPV clearance, underscoring the importance of prevention and successful treatment of bacterial vaginosis.

##  Condensation

Bacterial vaginosis is associated with increased odds for prevalent and incident HPV as well as delayed clearance among women in the HIV Epidemiology Research Study.

## Figures and Tables

**Table 1:** Characteristics of 756 HIV-infected and 380 HIV-uninfected women at enrollment to HERS.

Characteristic	HIV-infected *n* (%)	HIV-uninfected *n* (%)	*P* value
HIV risk category			
Injection drug user	391 (51.7)	184 (48.4)	0.29
High sexual risk	365 (48.3)	196 (51.6)	
Age			
<24	53 (7.0)	38 (10.0)	0.27
25–34	351 (46.4)	173 (45.5)	
35–44	310 (41.0)	144 (37.9)	
45+	42 (5.6)	25 (6.6)	
Race/ethnicity			
African American	457 (60.5)	200 (52.6)	<0.01
Caucasian	155 (20.5)	117 (30.8)	
Hispanic	135 (17.9)	59 (15.5)	
Native American or Asian	9 (1.2)	4 (1.1)	
Education			
<H.S.	342 (45.4)	141 (37.3)	0.01
H.S.	249 (33.0)	130 (34.4)	
>H.S.	163 (21.6)	107 (28.3)	
Number of live births			
0	126 (16.7)	79 (20.8)	0.22
1	171 (22.6)	78 (20.5)	
≥2	459 (60.7)	223 (58.7)	
Number of male sex partners in past 6 months			
0	187 (24.9)	56 (14.9)	<0.01
1	423 (56.3)	187 (49.7)	
≥2	141 (18.8)	133 (35.4)	
Lower genital tract infections			
Bacterial vaginosis	361 (47.8)	164 (43.2)	0.14
Vaginal *Candida* colonization	273 (36.2)	82 (21.6)	<0.01
*Trichomonas vaginalis *	96 (12.8)	40 (10.6)	0.29
*Chlamydia trachomatis *	8 (1.4)	5 (1.6)	0.98
*Neisseria gonorrhoeae *	6 (0.8)	1 (0.3)	0.28
Herpes simplex virus-2 antibodies	491 (66.7)	207 (56.0)	<0.01
Genital ulcers	60 (7.9)	16 (4.2)	0.02
Human papillomavirus			
Generic probe positive	482 (63.7)	109 (28.7)	<0.01
Untypeable	98 (20.3*)	33 (30.3*)	0.02
1 type	204 (42.3*)	56 (51.4*)	<0.01
2 types	96 (19.9*)	13 (11.9*)	
≥3 types	84 (17.4*)	7 (6.4*)	
Hepatitis C	443 (59.0)	178 (47.3)	<0.01
Recent crack cocaine or injection drug use	285 (37.7)	166 (43.7)	0.05
Cigarette smoker	566 (74.9)	294 (77.4)	0.35
HIV disease progression			
CD4+ lymphocyte count (cells/*μ*L)		n/a	
<200	130 (17.3)		
200–499	378 (50.3)		
≥500	243 (32.4)		
HIV RNA load (copies/mL)		n/a	
≥10,000	143 (19.3)		
3,000–<10,000	124 (16.8)		
500–<3,000	287 (38.8)		
<500	186 (25.1)		
Antiretroviral therapy		n/a	
None	500 (66.1)		
Sub-HAART^†^	256 (33.9)		

HIV: human immunodeficiency virus; RNA: ribonucleic acid.

*n* (%) = number of subjects and percent of HIV-infected or HIV-uninfected cohort.

*Percent of those who were generic probe positive.

^†^Sub-HAART was defined as self-reported use of any antiretroviral(s) that combined do not qualify as highly active antiretroviral therapy (HAART) according to the Department of Health and Human Services guidelines [26].

**Table 2:** Odds ratios for prevalent human papillomavirus among all HERS women and by HIV status.

Vaginal infection	HERS overall	HIV infected^1^	HIV uninfected^2^
	(8920 visits)	(5869 visits)	(3051 visits)
	aOR (95% CL)	aOR (95% CL)	aOR (95% CL)
Bacterial vaginosis	1.14 (1.04, 1.26)	1.15 (1.03, 1.29)	1.18 (0.98, 1.42)
*Trichomonas vaginalis*	∗		1.16 (0.91, 1.49)
Among *Candida *colonization+		1.05 (0.84, 1.32)	
Among *Candida* colonization -		0.75 (0.62, 0.90)	
Vaginal *Candida* colonization	∗		1.12 (0.93, 1.35)
Among *Trichomonas vaginalis*+		1.36 (1.05, 1.75)	
Among *Trichomonas vaginalis*−		0.97 (0.87, 1.08)	

HIV: human immunodeficiency virus.

aOR (95% CL) = adjusted odds ratio and 95 percent confidence limits.

Generalized linear mixed models with random intercept and slope based on the toeplitz matrix were adjusted for study site, risk behavior cohort, visit number, age group, race/ethnicity, cigarette smoking, illicit drug use, new or multiple male sex partners, and consistency of condom use. Model for HIV-infected women was also adjusted for CD4 group (<200, 200–500, and >500 cells/*μ*L) and logarithm of viral load.

^1^Statistical interaction between trichomoniasis and vaginal *Candida *colonization (*P* = 0.014).

^2^No statistical interactions detected at the *P* < 0.15 level.

*No odds ratios are presented for the overall HERS cohort because HIV status significantly modified the associations between trichomoniasis, vaginal *Candida *colonization, and HPV prevalence.

**Table 3:** Odds ratios for incident human papillomavirus among all HERS women and by HIV status.

Vaginal infection at prior visit	HERS overall	HIV infected	HIV uninfected
	(5587 visits)	(4038 visits)	(1549 visits)
	aOR (95% CL)	aOR (95% CL)	aOR (95% CL)
Bacterial vaginosis	1.24 (1.04, 1.47)	1.22 (1.00, 1.50)	1.22 (0.84, 1.77)
*Trichomonas vaginalis*	1.05 (0.84, 1.32)	1.11 (0.86, 1.44)	0.86 (0.53, 1.40)
Vaginal *Candida* colonization	0.97 (0.82, 1.15)	1.04 (0.86, 1.26)	0.75 (0.51, 1.11)

HIV: human immunodeficiency virus.

aOR (95% CL) = adjusted odds ratio and 95 percent confidence limits.

Conditional logistic regression models were adjusted for the following time-varying factors: illicit drug use, new or multiple male sex partners, and consistency of condom use since the last study visit. Model for HIV-infected women was also adjusted for CD4 group (<200, 200–500, and >500 cells/*μ*L) and logarithm of viral load.

**Table 4:** Hazard ratios for type-specific clearance of human papillomavirus among all HERS women and by HIV status.

Vaginal infection	HERS overall	HIV infected	HIV uninfected
	(1230 HPV infections)	(1016 HPV infections)	(214 HPV infections)
	aHR (95% CL)	aHR (95% CL)	aHR (95% CL)
*Trichomonas vaginalis*	1.06 (0.88, 1.27)	1.12 (0.91, 1.36)	1.00 (0.56, 1.78)
Bacterial vaginosis			
Among *Candida *colonization +	0.65 (0.51, 0.83)	0.71 (0.55, 0.94)	0.38 (0.16, 0.87)
Among *Candida *colonization−	0.96 (0.79, 1.15)	0.94 (0.75, 1.17)	1.25 (0.77, 2.05)
Vaginal *Candida* colonization			
Among bacterial vaginosis+	0.73 (0.58, 0.93)	0.76 (0.59, 0.99)	0.41 (0.17, 0.96)
Among bacterial vaginosis−	1.07 (0.89, 1.28)	1.00 (0.81, 1.24)	1.36 (0.85, 2.18)

HIV: human immunodeficiency virus; HPV: human papillomavirus.

aHR (95% CL) = adjusted hazards ratio and 95 percent confidence limits.

Wei, Lin, and Weissfeld extended Cox models were adjusted for study site, risk behavior cohort, visit year at onset of HPV infection, age group, race/ethnicity, cigarette smoking, new or multiple male sex partners, consistency of condom use, coinfection with another HPV type, and its cancer risk classification. Model for HIV-infected women was also adjusted for CD4 group (<200, 200–500, and >500 cells/*μ*L) and logarithm of viral load.

Statistical interaction between trichomoniasis and vaginal *Candida *colonization (HIV-infected model: *P* = 0.111; HIV-uninfected model: *P* = 0.018).
